# Monolithic Cylindrical Fused Silica Resonators with High Q Factors

**DOI:** 10.3390/s16081185

**Published:** 2016-07-28

**Authors:** Yao Pan, Dongya Wang, Yanyan Wang, Jianping Liu, Suyong Wu, Tianliang Qu, Kaiyong Yang, Hui Luo

**Affiliations:** College of Optoelectronic Science and Engineering, National University of Defense Technology, Changsha 410073, China; yaomeredithpan@hotmail.com (Y.P.); mywangdongya@126.com (D.W.); wxy1356238776@163.com (Y.W.); l_jianp@sina.com (J.L.); sywu2001@163.com (S.W.); muyiky@163.com (K.Y.)

**Keywords:** cylindrical resonator, fused silica, Q factor, chemical etching, annealing

## Abstract

The cylindrical resonator gyroscope (CRG) is a typical Coriolis vibratory gyroscope whose performance is determined by the Q factor and frequency mismatch of the cylindrical resonator. Enhancing the Q factor is crucial for improving the rate sensitivity and noise performance of the CRG. In this paper, for the first time, a monolithic cylindrical fused silica resonator with a Q factor approaching 8 × 10^5^ (ring-down time over 1 min) is reported. The resonator is made of fused silica with low internal friction and high isotropy, with a diameter of 25 mm and a center frequency of 3974.35 Hz. The structure of the resonator is first briefly introduced, and then the experimental non-contact characterization method is presented. In addition, the post-fabrication experimental procedure of Q factor improvement, including chemical and thermal treatment, is demonstrated. The Q factor improvement by both treatments is compared and the primary loss mechanism is analyzed. To the best of our knowledge, the work presented in this paper represents the highest reported Q factor for a cylindrical resonator. The proposed monolithic cylindrical fused silica resonator may enable high performance inertial sensing with standard manufacturing process and simple post-fabrication treatment.

## 1. Introduction

Coriolis vibratory gyroscopes (CVGs), especially those with axisymmetric shell resonators, are known for their outstanding capabilities of high accuracy, long durability, considerable reliability, low power consumption, short start-up time, high shock and acceleration resistance, etc. The well-known hemispherical resonator gyroscope (HRG) has claimed 30 million hours of continuous operation without a single mission failure [1]. Because this technology is complicated and rather expensive, researchers have tried to find acceptable compromise between cost and performance. As a simpler version, cylindrical resonator gyroscopes (CRGs) with metallic and piezoelectric cylindrical resonators have been intensively investigated [2,3,4,5,6,7,8,9]. The cylindrical resonator is the core component for CRGs with various applications where GPS may not be available, including inertial navigation systems, flight control systems, aircraft monitoring and maintenance, airborne platform stabilization, oil and gas platform stabilization, unmanned ground vehicles, north finding and automated guided vehicles, etc. There are tactical grade and industrial grade CRG products available on the market, such as Watson Industries Pro Gyro^®^ series [10] and the Innalabs 2000 and 1000 series [11]. These gyroscopes are readily applicable to medium and low accuracy fields such as platform stabilization, industrial control systems, rail-tilt control systems, short-term navigation, etc. However, the intrinsic disadvantage of metallic and piezoelectric cylindrical resonators is their low quality (Q) factors, which limits the overall performance of the gyroscopes. With excitation and detection elements, the piezoceramic cup resonator has a sense Q of only ~52 [12]. Resonators made of metallic alloys typically have Q factors up to 10^4^~4 × 10^4^ [7,13]. Notably, the Innalabs CVG25 resonators made from metallic alloys have shown a Q factor of 4 × 10^4^, which is so far the highest Q factor reported for metallic cylindrical resonators [7]. The Q factor of the combined cylindrical resonator with a fused silica shell and a metal plate is only about 6250 [14]. Designed by STM Co./ATSU [15], cylindrical resonators made of high-purity single-crystalline sapphire have shown promising Q factors of more than 10^7^. However, the sapphire material has intrinsic anisotropy and high stress, which will induce frequency mismatch for resonators. It is also worth mentioning that, while various micro cylinder or hemispherical cylinder resonators have been reported recently with Q factors of 5 × 10^5^~1.2 × 10^6^ [16,17,18], these resonators have much higher mode frequency, resulting in much shorter ring-down time. Although some notable experimental results have been reported, these technologies are still premature for high-accuracy gyroscopic applications.

The accuracy of CRGs operating in the force-to-rebalance mode is mainly determined by the Q factor and the natural frequency mismatch of the resonator [6]. Enhancing the Q factor is crucial for improving the rate sensitivity and noise performance of CRGs. Fused silica is an attractive material because of its intrinsically very low internal friction and high isotropy. Innalabs has predicted the Q factor of 25 mm-diameter resonators made from fused silica to be around 5 × 10^5^~7 × 10^5^ and the corresponding accuracy is a few of hundredths degree per hour in a temperature range of −40 °C to 50 °C, approaching inertial grade. However, no experimental results have been reported on monolithic cylindrical fused silica resonators up to now.

For the first time, we report a 25 mm-diameter monolithic cylindrical fused silica resonator with Q factor approaching ~8 × 10^5^ at a center frequency of 3974.35 Hz (the decay time is more than one minute) in moderate vacuum. In the following sections, the structure design of the resonators is first briefly presented. Then our non-contact experimental method to measure the Q factor of these resonators is demonstrated. Next the post-fabrication chemical and thermal treatment is presented and the Q improvement of six resonators is presented. Primary energy loss mechanism is analyzed and the corresponding solution is proposed.

## 2. Methods 

### 2.1. Resonator Structure

**Figure 1:**
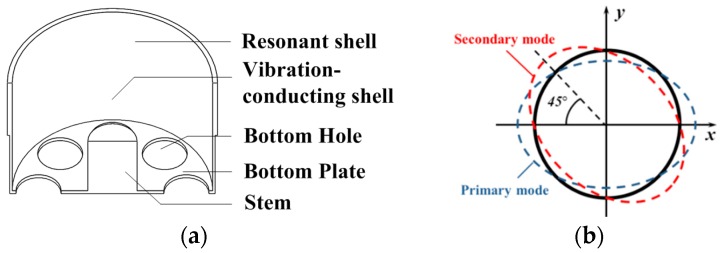
Schematic of: (**a**) the monolithic cylindrical fused silica resonator structure; (**b**) the *n* = 2 wineglass mode of an ideal cylindrical resonator.

**Figure 2:**
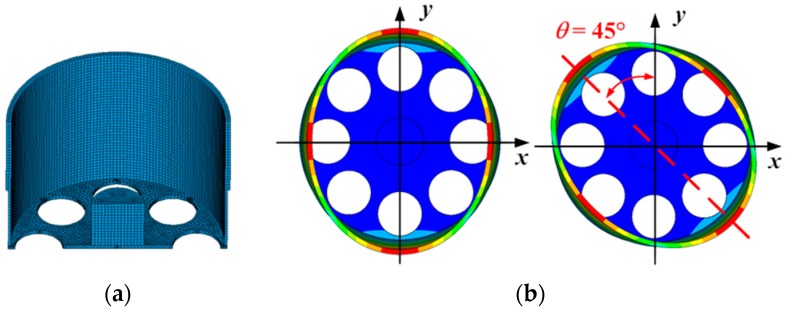
The finite element model and *n* = 2 wineglass mode: (**a**) The sectional view of the finite element model of the monolithic fused silica cylindrical resonator built with Ansys Multiphysics 14.0; (**b**) The simulated *n* = 2 wineglass mode of the resonator.

The structure of the monolithic cylindrical fused silica resonator is shown in Figure 1a. The resonator comprises a cylindrical resonant shell, a vibration-conducting shell, a bottom plate with eight holes equi-angularly distributed on, and a rigid stem on the bottom plate inside the cylindrical shell. The cylindrical resonator works at the *n* = 2 wineglass mode, with *n* referring to the number of circumferential node lines with respect to the axis of symmetry. For an ideal cylindrical resonator, the *n* = 2 wineglass mode actually includes two degenerate modes with the same natural frequencies and an angular interval of 45°, as depicted in Figure 1b, and they do not have a determined angular location around the axis of symmetry. These modes are often referred to as primary and secondary mode for excitation and detection mode, respectively. The finite element model was built using Ansys Multiphysics 14.0 software, as depicted in Figure 2a, and the simulated *n* = 2 wineglass mode was shown in Figure 2b. The cylindrical shell is the main vibrating body of the resonator, thus it demands extremely high machining precision. The vibration-conducting shell is considerably thinner than the cylindrical shell and the requirement on its machining precision is less stringent. The holes on the bottom plate ensure that the excitation and detection elements excite and detect the vibration mode of the resonator instead of membrane vibration of the bottom plate [19]. Furthermore, they may block the heat flow of the bottom plate while it deforms, thus decreasing the overall thermoelastic damping [20]. The rigid stem is used to fix the resonator, usually by gluing with epoxy or by indium bonding. In this design, the resonator can be excited and detected either by piezoelectric effect or by electrostatic force depending on the electrode design.

It should be pointed out that, our design was inspired by the structure first proposed by Chikovani et al. [6,7,19], based on which we fabricated the monolithic fused silica resonator, which is different from the work reported by Xiang et al. [13,14,21], in which they investigated resonators made of metallic alloys and resonators with fused silica shells glued onto metallic disks, which will hardly result in high Q resonators for high-accuracy applications because of the material used and gluing procedure. In addition, our stem design was different from reported resonators for the simplicity and better precision of manufacturing.

### 2.2. Manufacturing Process

**Figure 3:**
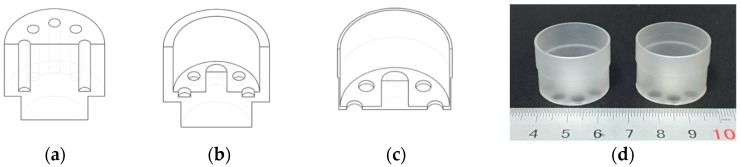
Schematic of the manufacturing process of the monolithic cylindrical fused silica resonator: (**a**) The frock was roughly ground and holes were formed during the first step; (**b**) The inner surface was finely finished during the second step; (**c**) The outer surface and the bottom plate were finished during the last step; (**d**) The photo of monolithic cylindrical fused silica resonators.

A series of cylindrical fused silica (Suprasil-311) shell resonators of varied sizes were manufactured. The manufacturing process was typical deep grinding consisting of four steps. First, the fused silica block was roughly ground to a preliminary cylinder and eight holes were formed, as shown in Figure 3a. Then the inner and outer surface was finely ground, as shown in Figure 3b. The last step was to grind the outer surface and the bottom plate to the designed size, as shown in Figure 3c. As the shell is very thin, a cylinder frock was used to avoid cracking. In this paper, six resonators were considered. The overall dimensions of the six resonators were measured by a Werth Messtechnik GmbH (Giessen, Germany) measurement microscope, and the bottom thickness was measured by a micrometer. The resonator radius R is 12.5 mm and the diameter of the bottom hole is 3.5 mm for resonator CR01 and CR02 and 5.5 mm for the rest resonators. The main structural parameters were listed in Table 1, where H, h and L, l stand for the thickness and length of the resonant shell and the vibration-conducting shell, respectively; h_b_ and d denote the thickness of the bottom plate and the diameter of the bottom hole, respectively; d denotes the diameter of the bottom hole. Due to our special support design, the resonator can be finished with only one clamping, resulting in high manufacturing accuracy especially regarding cylinder rim roundness and resonant shell concentricity, which were within 3 μm and 1 μm, respectively. As a result these as-fabricated resonators have small natural frequency split (typically 0.031 Hz–0.555 Hz without any special balancing procedure), which is also crucially important to the performance of the gyroscope in addition to a high Q factor [22].

### 2.3. Testing Apparatus

**Figure 4:**
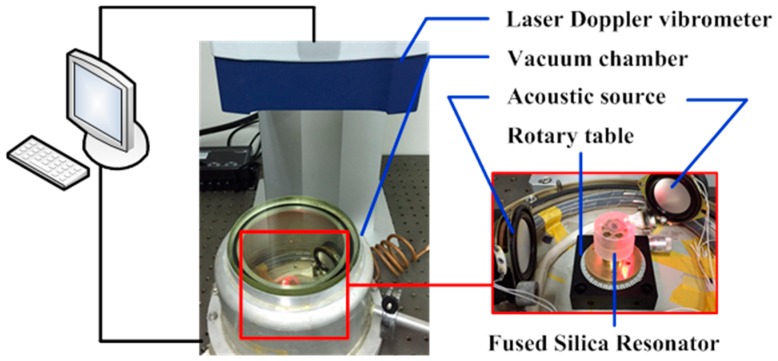
The experimental setup includes a Polytec laser Doppler vibrometer, a vacuum chamber, an acoustic source and a rotary table. This setup allows non-contact characterization of the resonator.

The experimental setup is shown in Figure 4, which consists of our designed vacuum chamber and a laser Doppler vibrometer (Polytec, Irvine, CA, USA). They were placed on an optical table to avoid environmental vibrations. The rigid stem of the resonator is clamped with our designed fixture and together they are mounted on a rotary table with epoxy glue (EPO-TEK 301, EPOXY TECHNOLOGY, Billerica, MA, USA). The resonator is excited by an acoustic source and its vibration detected by the laser Doppler vibrometer, which enables non-contact characterization. The resonator and the acoustic source are placed in the vacuum chamber, where the air damping can be greatly reduced, and the optical window on the vacuum chamber allows laser Doppler vibrometry characterization. This method measures the actual performance of the resonator itself, eliminating electrical damping and other damping that may occur when driving and sensing elements were attached on the resonator.

It is known that, in practice, due to material anisotropy and manufacturing errors, the resonator has a natural frequency mismatch and a pair of principle axes of vibration. Moreover, in practice the Q factor of the resonator is not circumferentially homogeneous. The change of the excitation direction will further lead to the variation of the Q factor. However, with repetitive experiments we find that, the Q factor of the antinode is relatively stable. For example, to observe the stability of the antinode Q factor, we measured the Q factor variation by changing the excitation direction and measure the antinode Q factor of one resonator. The average value, standard derivation and relative variation were calculated, and the results are listed in Table 2.

**Figure 5:**
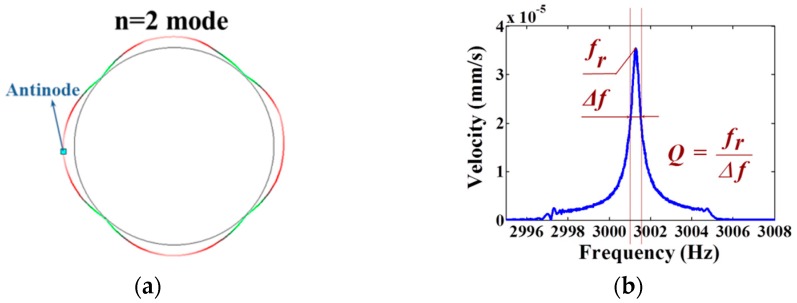

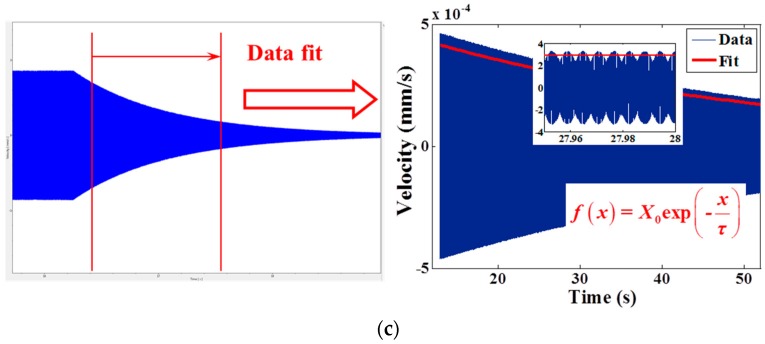
Exemplary steps of the Q factor test. (**a**) First the vibration pattern was recorded and the antinode of *n* = 2 wineglass mode was selected to be the subsequent testing point; (**b**) The frequency response of the monolithic cylindrical fused silica resonator was measured and the Q factor of the *n* = 2 wineglass mode was calculated by the Polytec software; (**c**) The time signal was recorded and the data was fitted in MATLAB to calculate the ring-down time of the *n* = 2 wineglass mode of the resonator. The enlarged picture shows the beating of the signal due to the sampling process.

Results show that, a 6-degree change of the excitation direction leads to only 0.5% variation of the antinode Q factor, and a 25-degree change of the excitation direction results in no more than 2% variation of the antinode Q factor. Therefore, the Q factor testing procedure is carefully designed as follows. First the resonant frequency *f_r_* of the resonator is sought out by exciting the resonator with a sweeping signal in a relatively wide frequency range. Then the resonator is excited by a sinusoidal signal with its resonant frequency, during which the vibration pattern is recorded and the antinode is determined, as shown in Figure 5a. Then the resonator is again excited by a sweeping signal in a narrower frequency range, so as to have a better measurement resolution, and the vibration at the antinode is recorded. 

The Q factor of the resonator is then calculated by the Polytec software according to:

(1) $$Q=\frac{\Delta f}{f_{r}}$$

where *Δf* is the 3-dB bandwidth of the frequency response of the resonator, as shown in Figure 5b. The frequency resolution selected is 0.012 Hz. For much higher Q, the resonant characteristics are measured using the ring-down method, i.e., the shell is attenuated at its resonant frequency and the time constant τ for the vibration amplitude to decay by 1/e is measured. The resonant frequency is determined the same as in the frequency response method, and the sinusoidal signal with the resonant frequency is used to excite the resonator. When the wineglass mode vibration of the resonator is stable, the signal is shut down and the resonator is allowed to freely vibrate with damping. The data is recorded by the laser Doppler vibrometer, an appropriate length of which was acquired and fitted exponentially in MATLAB, as shown in Figure 5c. Q is then calculated by:

(2) $$Q=\pi f_{r}\tau$$

Specifically, the data recorded is typical exponentially damped sinusoidal signal. We have calculated the frequency of the signal by fast Fourier transform, and found the difference between the calculated frequency and the excitation frequency was on the order of 10^−3^ Hz. Therefore we use the frequency of the excitation signal obtained during frequency sweeping as a good approximation to calculate Q in the ring-down time method. We use cubic spline interpolation to derive the upper envelope of the signal, and then fit the envelope with exponential function by least square nonlinear fitting. As shown in the enlarged picture in Figure 5, the decay curve has beating with a period of roughly 6 × 10^−3^ s, which is caused by the insufficient sampling frequency provided by the FFT mode of the laser Doppler vibrometer. The error caused by this effect should be on the order of 10^−3^ s.

### 2.4. Q Factor Improvement

Q is known to be affected by many different damping mechanisms, including surface loss *Q_surface_*, material loss *Q_material_*, air damping *Q_air_* and anchor loss *Q_anchor_*, etc. The total Q is limited by the greatest damping as in [23]:

(3) $$\frac{1}{Q}=\frac{1}{Q_{surface}}+\frac{1}{Q_{material}}+\frac{1}{Q_{air}}+\frac{1}{Q_{anchor}}+\frac{1}{Q_{etc.}}$$

The optimization of Q factor requires different damping mechanisms being individually addressed. While air damping is the most dominant effect at atmospheric conditions, it can be easily reduced by operating the resonator in vacuum. The proposed experimental apparatus enables the measurement of Q in moderate vacuum (the pressure was ~10^−2^ Pa measured by an ionization gauge near the molecular pump, therefore in the vacuum chamber the pressure is higher, especially when the pump was shut down and there were organic chemicals inside. The pumping time was set to half an hour to ensure the same moderate vacuum conditions). Anchor loss is caused by energy transferring from resonator to substrate though the anchor, and is minimized by balancing the resonator and careful design of the fixture. To ensure the condition of the fixing, a torque wrench was used in the test and the fixing torque was set to 0.05 N·m.

Surface loss is mainly caused by a defect layer formed during the grinding and polishing of the resonators. The surface of the fused silica resonator after mechanical grinding is subjected to subsurface damage (SSD), which refers to the residual fractured and deformed material in the near surface region. This layer of deformed and fractured material exhibits substantially different mechanical behavior, particularly a significantly higher internal friction. It was previously studied in optical fine machining that even by meticulously optical polishing, there would still be SSD remained. The surface loss can be effectively reduced by chemically removing the surface layer [24,25,26,27]. 

The material loss consists of several loss mechanisms, including thermal elastic damping and microscopic material defects, etc. The upper limit on the attainable quality factor is determined by material and geometric structure of the resonator [20]. However, the Q on the order of 10^7^ is easily attainable for bulk materials of the high purity synthetic fused silica [28], thus in millimeter-diameter range other loss needs to be suppressed first before considering thermoelastic damping. Other material loss is usually minimized by using a high purity and isotropic material with low coefficient of thermal expansion.

In addition, annealing can improve the Q factor through the relieving of internal stress and the removing of surface contaminants [29,30]. Post-fabrication annealing has been employed in various MEMS resonators [31,32], improving their Q for more than an order of magnitude, and more recently in micro shell resonators [33,34] whose Qs were also moderately improved. With the above considerations in mind, we performed both chemical and thermal treatments to improve the quality factor of the proposed monolithic cylindrical fused silica resonator.

## 3. Results

### 3.1. Chemical Etching

The chemical etching procedure proposed in [25,26] was employed, and resonators CR01, CR05 and CR06 were chemically etched. For each etching round, the etching depth was measured by the Werth Messtechnik GmbH measurement microscope and the surface roughness was measured by a Taylor Hobson (Berwyn, PA, USA) profiler.

**Figure 6:**
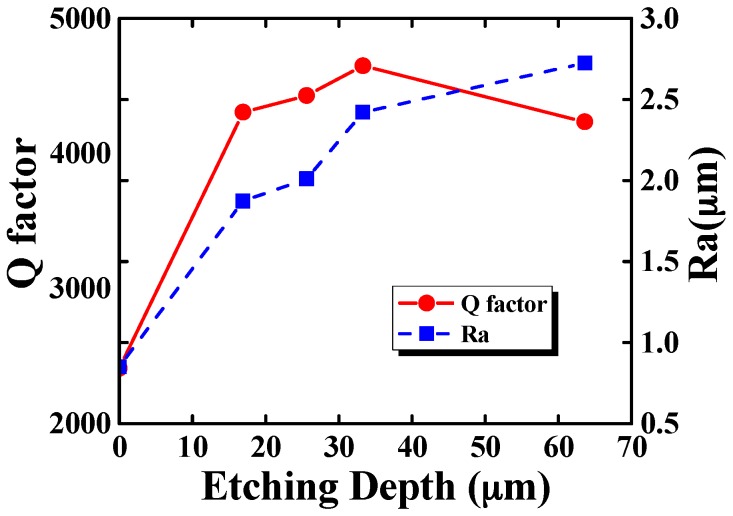
The surface roughness and Q factor change with etching depth of resonator CR01.

**Figure 7:**
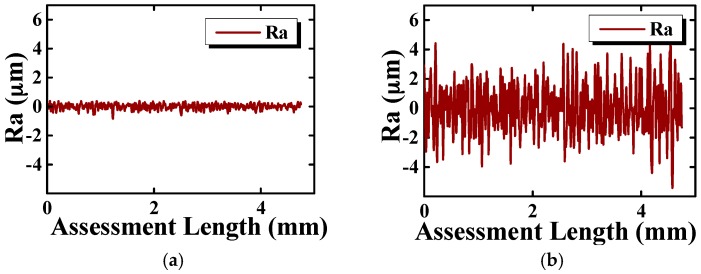
Surface roughness of CR01. (**a**) The surface roughness plot before chemical etching; (**b**) The surface roughness plot after chemical etching.

Resonator CR01 was first etched in a solution of 10:1 in volume ratio 40 wt % NH_4_F and 49 wt % HF in ambient temperature for two hours followed by three one-hour periods. The resonant frequency and Q factor for the *n* = 2 mode were measured five times continuously in each round and the relative errors were within 10 ppm and 2%, respectively. The results of the chemical etching process were listed in Table 3 and depicted in Figure 6. The parameters considered were the etching depth, the surface roughness, the resonant frequency and Q factor of the resonator. Results showed that the Q factor improves significantly, especially after the first etching round, when the Q factor improves by 78.7% and the resonant frequency decreased by 2.40%, while the etching depth is 16.9μm. Results also showed that the surface roughness deteriorates with each etching period. The surface roughness of resonator CR01 before and after chemical etching is depicted in Figure 7, which clearly shows the degradation of the resonator surface. The Q drop in the last experimental period suggests that further degradation of the surface roughness or other unknown factors might cause extra damping. According to the results measured in atmospheric condition, it seemed that the optimum etching depth should be around 33.3 μm, where the Q factor of resonator CR01 in air was improved by 92.9%. Later measurements in moderate vacuum suggested otherwise.

**Figure 8:**
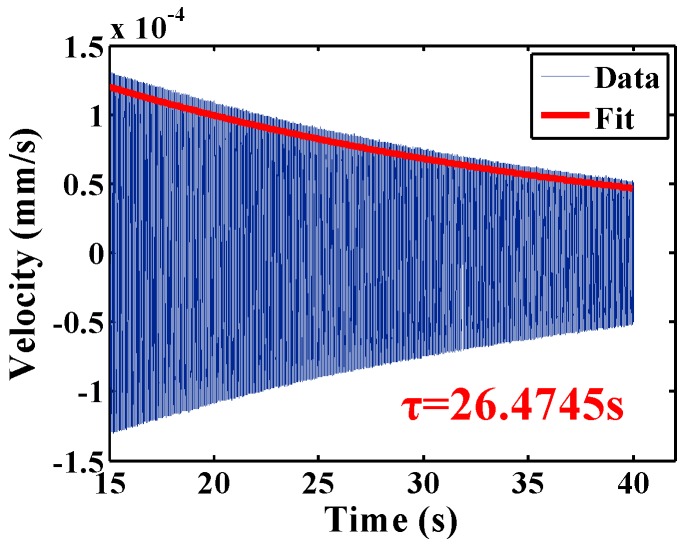
Ring-down time experiment of resonator CR01 in moderate vacuum shows *τ* = 26.4745 s at *f* = 5353.29 Hz, giving a Q factor of 445,245.

To reduce the air damping, the resonator CR01 was then tested in moderate vacuum. The ring-down time signal and the exponential fit result were plotted in Figure 8. The time constant of the resonator is 26.4745 s and the corresponding Q factor is 445,245, which 104.2 times greater than that in air. Later experiments revealed that the Q factors of the resonators in moderate vacuum before chemical etching were within ~2 × 10^4^, therefore the Q improvement here is mainly due to chemical etching.

### 3.2. Annealing

We performed annealing on resonators CR02, CR03 and CR04, which were annealed at 1050 °C for 12 h in an electric vacuum furnace. All three resonators were annealed in ~10^−2^ Pa vacuum to protect the resonator surface from being contaminated and avoid surface defect during annealing [30]. The resonators were heated at a rate of 5 °C/min, then the furnace temperature was preserved at 1050 °C for 12 h. Then the two resonators were cooled to 800 °C at a rate of 1 °C /min to ensure no extra internal thermo-stress induced, followed by free cooling to room temperature.

**Figure 9:**
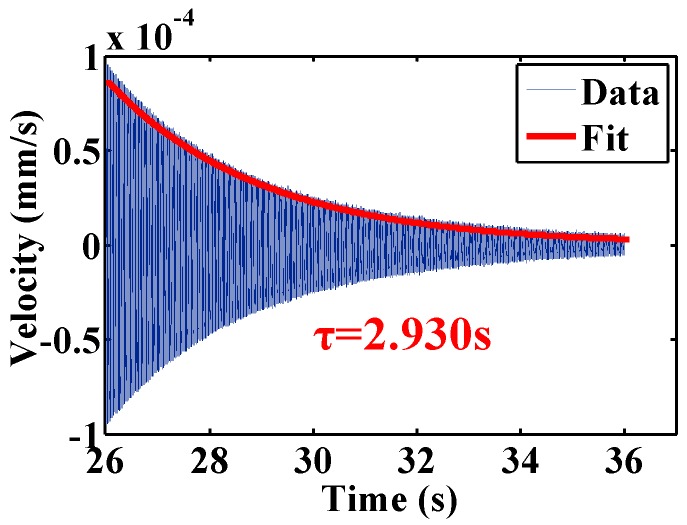
Ring-down time experiment of resonator CR04 in moderate vacuum shows *τ* = 2.930 s at *f* = 4031.02 Hz, giving a Q factor of 37,105.

Q factors for *n* = 2 mode were tested and the results are listed in Table 4. All three of the resonators experienced a small increase in resonant frequency as well as a notable improvement of Q. Their resonant frequencies were increased by 0.16%, 0.28% and 0.18%, respectively, while their Q factors were increased by 88.1%, 51.2% and 61.4%, respectively. To reduce the air damping, the resonator CR04 was then tested in moderate vacuum. The ring-down time signal and the exponential fit result were plotted in Figure 9. The corresponding Q factor was 37,015, which increases 3.8 times than that in air. Compared with the Q improvement by chemical etching, which is 104.2 times, the improvement brought by annealing is relatively small. 

**Figure 10:**
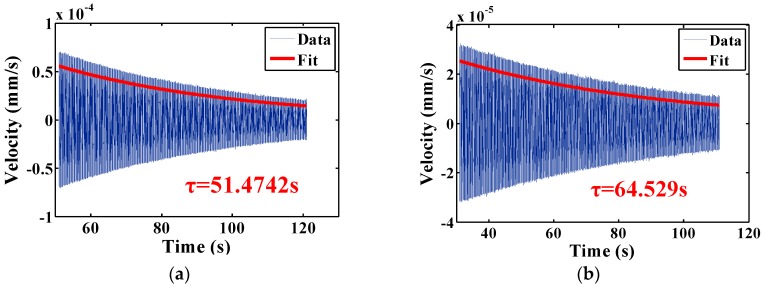
Ring-down time experiment of resonator CR05 and CR06 in moderate vacuum. (**a**) Resonator CR05 shows *τ* = 51.4742 s at *f* = 4453.49 Hz, giving a Q factor of 720,178; (**b**) Resonator CR06 shows *τ* = 64.529 s at *f* = 3974.35 Hz, giving a Q factor of 805,898.

Enlightened by earlier results, we focused our efforts on chemical etching. Resonators CR05 and CR06 were etched in a slightly different recipe with heating to accelerate the etching process. Before and after etching each resonator was tested in both atmospheric conditions and in moderate vacuum. The results were listed in Table 5, where *f_r_* and Q represent resonant frequency and Q factor in air while *f_r_’* and Q*’* represent resonant frequency and Q factor in vacuum. The etching depths of resonator CR05 and CR06 were 91μm and 106 μm, respectively. Their Qs in air were 7647 and 9115, respectively, improved by 93.1% and 99.1% compared with initial Q factor in air. Their Q factors in vacuum were ~7 × 10^5^ and ~8 × 10^5^, respectively, improvement of 37.8% and 47.7% compared with the initial Q factor in vacuum. The testing results in vacuum showed that, in contrast to earlier results, the optimum etching depth is likely more than 100 μm. The ring-down time signal and the fitting results were depicted in Figure 10. Notably, resonator CR06 show a Q factor approaching ~8 × 10^5^ at the center frequency of 3974.35Hz, which is more than that predicted by Innalabs [7].

## 4. Discussion

Results with chemical etching showed that our resonators experience dramatic Q factor improvements after surface treatment. This confirms that surface loss is indeed important, as suggested by Lunin [35], in macroscopic fused silica cylindrical resonators fabricated by grinding, in contrast with previous studies on bulk materials, which suggest that surface loss is not so significant [28,36]. This is likely due to the fact that our resonators have a large surface-to-volume ratio and their surfaces are mostly ground curve surfaces. In addition, in contrast with the case with micro resonators [37], the surface roughness alone does not have a clear relationship with Q, suggesting that the micro-cracks which cannot be revealed by surface roughness are most likely the main source of surface loss. Therefore the improvement of Q factors by chemical etching is likely due to the removing of the hydrated and contaminated surface layer suggested in [27].

We compared our results with those published by Lunin et al. [35] using the simple model proposed in [28]. We used the same nomenclature as in [28], denoting total surface area as *S_t_*, curved surface area as *S_c_*, and total volume as *V*. The total-surface-to-volume ratio of the bulk cylinder is *S_t_/V*~0.22 mm^−1^, and the curved-surface to volume ratio is *S_c_/V*~0.20 mm^−1^. For our resonator, they are calculated as follows:

(4) $$\frac{S_{t}}{V}\sim 2.92\;{\mathrm{mm}}^{-1},\;\frac{S_{c}}{V}\sim 2.46\;{\mathrm{mm}}^{-1}$$

From Equation (3), our cylindrical resonator has approximately 13.2 times more total surface and 12.3 times more curved surface relative to the bulk cylinder. Therefore, we would anticipate a maximum Q of 2.27 × 10^5^ before etching for our resonator, or 2.43 × 10^5^ if considering only the curved surface, and one would expect a maximum Q of 1.51 × 10^6^ after etching, or 1.62 × 10^6^ if considering only the curved surface. The fact that the Q factors of our resonators were below this value is likely due to the residual air damping and possible anchor loss.

In addition, the mechanism of Q factor improvement due to annealing is commonly considered related with internal stress relieving [24,33], surface quality improving and material removing [16,33], etc. Various studies showed that annealing improves the Q factors of microresonators significantly [16,33,34], and also moderately improves Q factors of bulk fused silica material [30,36]. However, it does not, at least in our resonator structure, contribute to a marked Q factor improvement. For our specific resonator, Q factor improvement by chemical etching is one order of magnitude higher than that by annealing, which suggested the surface loss is the predominant loss after the reduction of air damping. Further studies are still necessary on understanding the mechanism of surface loss.

It should be noted that, by “monolithic” we mean that our resonators are one-piece fused silica resonators, contrary to metallic cylindrical resonators or combined fused silica resonators as in literature [6,7,13,14]. We also acknowledge the fact that miniaturization is the future trend; however, our target at this stage is a macroscopic high-accuracy gyroscope for high-precision inertial applications. Although chemical and thermal treatments are widely used in literature to improve the resonator Q factor [25,26,27,30,31,32,33,34,35,37,38], no one has specially studied the effects of chemical and thermal treatments on monolithic cylindrical fused silica resonators. For example, in ref. [25], the distribution of subsurface damage in fused silica was studied systematically, specifically on the depth and morphology of subsurface fractures, aiming at correlating subsurface damage with abrasive size and load of the grinding process. However, the relationship between mechanical Q factor and the distribution of subsurface damage was not mentioned. In ref. [38], chemical etching is used to shape the LiNbQ_3_ disk resonator, while thermal treatment is used to improve the surface quality. However, the material, structure and application of the resonator are totally different from ours. Actually, the optimized process and effects of chemical and thermal treatments depend on the material and structural parameters of specific resonators, which is why there are many studies on chemical and thermal treatments to reduce the mechanical energy loss. We believe that, for our proposed resonator, it is of practical significance to study the suitable treatment procedures accordingly. As various literatures have indicated, with different materials and manufacturing processes, the effects of chemical and thermal treatments might be different [33,34,37] and optimum procedures need to be developed, in which we shall devote more effort. 

In conclusion, for our monolithic cylindrical fused silica resonators, the air damping and the surface loss are the two major losses in atmospheric conditions. The air damping limits Q factors to be below ~1 × 10^4^ even after the significant decreasing of surface loss by chemical etching. Therefore in practice resonators must work under appropriate pressure conditions. After the reduction of air damping by measuring the resonator in moderate vacuum, other losses limit Q to be within ~2 × 10^4^. After the reduction of internal loss and air damping the remaining losses limit Q to be around ~4 × 10^4^. After the reduction of surface loss and air damping, the Q factor approaches ~8 × 10^5^. Therefore, with standard fabrication process and simple post-fabrication chemical treatment, the resonator Q is more than sufficient for medium accuracy gyroscope applications. 

However, our test apparatus limits the testing pressure to allow the energy transfer from acoustic source to the resonator. The real Q might still be higher than the present results suggest, therefore our future work shall include developing suitable excitation methods to excite resonators under higher vacuum. For even higher Q, functional anchor structure must be carefully designed to reduce the anchor loss. Furthermore, the Q factor may be further improved by understanding the mechanism of surface loss and internal-stress related loss, and by carefully designing combined procedure of chemical and thermal treatment. When all other losses are minimized, thermoelastic damping associated with geometric structure needs to be considered. We are also developing gyroscopes based on the proposed resonators with piezoelectric and electrostatic transducing. The sensibility and stability characteristics will be presented in the future.

## 5. Conclusions

In this paper, the structure of the resonator is first briefly introduced, which is followed by the demonstration of the post-fabrication experimental characterization of the resonator Q factor. The experimental Q improvement is presented, including both chemical and thermal treatments. Results showed that the Q factor can be improved by more than an order of magnitude through chemical etching alone, while the Q improvement by annealing is small compared with chemical etching. Therefore for our monolithic cylindrical fused silica resonators, the surface loss is the major loss after the reduction of the air damping.

In addition, we report the first 25-mm diameter monolithic cylindrical fused silica resonator with a Q factor approaching ~8 × 10^5^ at the frequency of 3974.35 Hz. The high Q factor is enabled by fused silica material with low internal friction and high isotropy, as well as a simple chemical etching process. To the best of our knowledge, the Q factor presented in this paper is the highest reported Q factor for a cylindrical resonator at 25 mm diameter. The monolithic cylindrical fused silica resonator may enable precision inertial sensing applications with standard manufacturing process and simple post-fabrication chemical treatment.

## Figures and Tables

**Table 1:** Main geometric sizes of the considered resonators.

Devices	*H*	*L*	*h*	*l*	*h_b_*	*d*
CR01	1.1730	9.8364	0.2643	8.1657	0.495	3.45
CR02	1.1730	9.8073	0.2762	8.2266	0.559	5.43
CR03	0.6620	9.8067	0.3405	8.0389	0.950	5.52
CR04	0.7924	9.9008	0.3025	8.0124	1.105	5.51
CR05	0.8970	10.2848	0.3035	8.0401	1.100	5.53
CR06	0.9965	10.0610	0.2995	8.6200	0.625	5.53

**Table 2:** The antinode Q factors of the resonator while changing the excitation angle.

Average	STD	Variation	Angle (°)	Q Factor
9965.71	46.89	0.47%	0	9896
			1	9951
			2	9921
			3	10,024
			4	10,001
			5	10,004
			6	9963
9728.33	174.77	1.8%	0	9896
			5	9951
			10	9921
			15	10,024
			20	10,001
			25	10,004

**Table 3:** The etching depth, change of surface roughness and resonant frequency, and the Q factor improvement of Resonator CR01 by chemical etching.

Time (hour)	Depth (μm)	Ra (μm)	*fr* (Hz)	Q factor
0	0	0.850	5556.1	2410
2	16.9	1.874	5423.0	4306
3	25.6	2.010	5401.5	4430
4	33.3	2.422	5378.3	4650
5	63.6	2.724	5353.3	4234

**Table 4:** The comparison of Q factors and mode frequency of CR02, CR03 and CR04 before and after annealing.

Device	Before Annealing		After Annealing		Variation	
	*fr* (Hz)	Q factor	*fr* (Hz)	Q factor	*fr* (Hz)	Q factor
CR02	5556.4	1896	5565.2	3567	0.16%	88.1%
CR03	3456.4	4339	3466.2	6562	0.28%	51.2%
CR04	4013.9	4784	4021.3	7725	0.18%	61.4%

**Table 5:** Q factors of CR05 and CR06 before and after etching both in air and in moderate vacuum.

Device	Before Etching				Depth (μm)	After Etching			
	*fr* (Hz)	*Q*	*fr’* (Hz)	*Q’*		*fr* (Hz)	*Q*	*fr’* (Hz)	*Q’*
CR05	4814.7	3960	4821.2	18,147	91	4442.2	7647	4453.5	703,318
CR06	4459.7	4577	4471.3	16,320	106	3964.1	9115	3974.5	794,148
